# Memoirs of the Royal Medical Society at Paris

**Published:** 1782

**Authors:** 


VII. *Memoirs of the Royal Medical Society at Paris.* (Continued from p. 160).

*Surgery.*—The firft article under this head is the Society's *Report concerning the Operation of Caftration as practifed for the radical Cure of Hernias.* This paper relates to an enormity of which Heifter, Dionis, and other writers have repeatedly complained. The Breflaw phyficians affure us (*Hiftor. Morb. Vratiflav.*) that in their city a fingle operator in this way mutilated upwards of 200 children. The fame horrid practice, according to Haller, prevails in Switzerland. The intendants of Paris and Languedoc, ftruck with the frequency of it in their feveral diftricts, applied to the minifter, and the latter requefted the fociety to direct their attention to this fubject. Some of thefe operators, we are told, make no difficulty of taking out both teſticles when there is a double hernia. From an enquiry made by order of the biſhop of Papoul in Languedoc it appears, that upwards of 500 children have lately been mutilated in that ſingle dioceſe. Meſſieurs Poulletier de la Salle, Andry, and Vicq D'Azyr (the authors of the report) in order to remedy this grievance adviſe the putting in force certain good but obſolete laws, which inflict proper puniſhment on ignorant perſons who undertake operations.——2. *Remarks on the Cure of Ulcers by the vacillatory Motion of a Burning-glaſs.* M. la Peyre, a ſea ſurgeon, relates ſeveral caſes of foul ulcers with callous edges, which were cured by this practice. He applies the lens ſeveral times in a day, carrying it over the whole ſurface of the ulcer till a conſiderable degree of heat is excited. ——3. *An Account of a Cancer of the lower Lip, cured in three weeks by means of a Burning-glaſs. By* M. le Comte, Surgeon at Arcueil. The pain excited by this practice, we are told, is much eaſier to ſupport than that occaſioned by the actual cautery. After the cauteriſation each day, our author applied a compreſs dipped in ſpirit of wine, and the eſchar was generally thrown off in twenty-four hours, leaving the wound of a vermilion colour. The cure cure was effected without any loss of substance. M. le Comte observes, that the practice of insolation was adopted by the ancients, who were accustomed to expose dropsical patients to the rays of the sun. He adds that the lens may be used with advantage even in winter, provided there is a little sun-shine.—4. *A new Method of remedying Wounds of the Arteries. By* M. le Comte, Student of Surgery. This is an ingenious contrivance. It consists of a quill slit up on one side, so as to surround the artery, and lined with a fine ribbon, the ends of which serve as ligatures. The author has applied it to the carotid and crural arteries of several dogs and sheep, with success. The society having appointed a committee to ascertain the merits of this invention, they laid bare the crural artery of a dog for a considerable length, and after introducing it into the tube, punctured the vessel with a lancet. They then tied the ends of the ribbon, and the hæmorrhage stopt. On the third day they withdrew the tube, but not without difficulty, as the integuments were much inflamed, and the ends of the ribbon were glued together by the pus. The animal was preserved three weeks, and then was killed; but about eight days before his death a similar operation,

 though though with a larger incision, was performed on the left crural artery. After death both the arteries were injected. The right was completely filled with wax, but was a little contracted at the place of the operation. In the left the wax did not pass beyond the incision, the coats of the vessel being thickened, and its cavity obliterated. In two other dogs large incisions were made in the same artery, and in both the cavity was obliterated. The society promise to continue their inquiries on this subject.——5. *An Account of a full-grown Fœtus extracted from an Abscess of the Abdomen. By* M. Debois. The woman, who is the subject of this case, was in her fourth pregnancy, and had gone her full time. The waters were evacuated, and the midwife said she felt the head of the child. At this period of the labour the patient complained of a sudden and most violent pain, which, after continuing for a short time, went off, and she then complained only of a heavy weight in the hypogastric region. Two months after this she began to be attacked with a throbbing pain of the abdomen, and a suppuration took place. The abscess opened in two places, and the discharge from it was extremely offensive. She was now much reduced by hectic fever, colliquative sweats, and diarrhœa.

rhœa. At the end of three months ſhe was carried to the Hotel Dieu at Paris, where the abſceſs was dilated, and the bones of a full-grown fœtus extracted from the wound. The diſcharge for ſome time after this was ichorous and fœtid, but in about four months ſhe recovered her ſtrength and fleſh, and left the hoſpital in good health. She has ſtill, however, a fiſtulous ſore through which there is ſometimes a diſcharge of fæces. Our author has met with accounts of two caſes ſimilar to this. One of them is by Littre, in the *Mem. de l'Acad. des Sciences*; the other is in the *Journal Encyclopedique de Bouillon* for 1777.——6. *A Caſe in which a Child's arm was extracted from an Abſceſs ſome time after Delivery.* *By* M. Bouillon, Phyſician at Mortain. A woman at the full time was delivered of a dead child; but one of its arms was torn off, and remained *in utero.* The patient continued to feel pains in the abdomen, a conſiderable degree of fever enſued, and an inflammatory tumour appeared in the hypogaſtric region which ſuppurated. The bones of the child's arm were extracted from this abſceſs, and the woman recovered.——7. *An Improvement in the Operation of Laryngotomy.* *By* M. Vicq D'Azyr. The thyroid and cricoid cartilages are are ſeparated anteriorly by a ſmall triangular ſpace, which may always eaſily be found, however much the neck may be ſwelled. This is the place in which our author adviſes the operation to be performed. He has tried it in dogs with ſucceſs.

*Anatomy.*—1. *An Account of the growth of a horny Subſtance. By* M. Gaſtellier. The ſubject of this paper is a woman 97 years old, who at the age of 83, having a number of wens on her head, began to perceive an extraordinary protuberance on the lower part of her left temple, which increaſed rapidly, and acquired a horny conſiſtence. This excreſcence was ſawed off by a ſurgeon, but it ſoon grew again. An engraving of one of theſe horns is given of its natural ſize. It is about three inches long, as large as a little finger, and twiſted. Its baſis adhered to the ſkin only, not to the bone. Similar caſes are mentioned by Ingraſſius, Hildanus, and Thomas Bartholine.—2. *An Account of a natural Separation of the Bones of the Pelvis. By* M. Souquet, Phyſician at Boulogne. The patient, whoſe caſe is here related, is an Engliſh lady, of the name of Harris, who, in her twenty-fourth year and third pregnancy, had a very long and painful ful labour. At length the offa pubis feparated nine tenths of an inch, and fhe was then immediately delivered. A *crepitus* was perceptible on the leaft motion, a proper bandage was applied, the bones united again in about three months, and fhe has fince had five natural labours.—3. *A Defcription of a monftrous Fœtus. By* M. Vicq D'Azyr. This child, inftead of a mouth, had only a very fmall round opening, terminating in a conical pedicle. It had no eyes, nofe, or palate, and only one ear. The reft of the body was perfect.——4. *A Cafe in which the frontal Nerve was wounded. By* the fame. A young furgeon, in fencing, loft his fight by the ftroke of a foil; on examining the wound it appeared that the foil had ftruck on the fiffure which gives paffage to the frontal nerve, fo that the nerve was almoft entirely divided. M. Vicq D'Azyr has fince divided this nerve in quadrupeds, but without producing any fuch effect.——5. *An Account of the fingular Difeafe of the Widow Melin, commonly called* la Femme aux Ongles. *By* M. Saillant. This woman, who was born in 1730, dated her complaints from the birth of her fecond child, which happened in 1752. The lochia were fuppreffed on the third day after her delivery. On the 9th fhe went out, got wet, and was foon after after ſeized with a violent head-ach, loſs of appetite, and pains in her knees, attended with conſiderable ſwelling and inflammation. Theſe complaints reſiſted a variety of medicines for the ſpace of ten months, at the end of which time the extenſor tendons of her foot contracted, and ſhe was obliged to keep to her bed. Abſceſſes formed about the coccyx, and the ſuppuration laſted a year, attended with long and frequent ſyncopes, preceded by cold ſweats. The exceſſive pain of her head occaſioned the loſs of her eye-ſight, and by degrees all the muſcles of her lower limbs became contracted. During the three firſt years of this diſeaſe ſhe got no ſleep; but at the end of that period ſhe ſlept for five hours, her menſes, which had ceaſed all that time, re-appeared, and her pains leſſened. About this time foul ulcers began to form about the ends of her fingers, and her nails grew long, fleſhy, and ulcerated. She had copious ſweats, and an almoſt inceſſant ſalivation. Her bones became fragile and incapable of reſiſting the flexor tendons, ſo that all her limbs were bent inwards except her right arm, which became twiſted through its whole length. In the mean time her general health ſeemed to be mended, and ſhe grew fatter; but in 1758 ſhe was attacked with with peripneumonia notha, and the year following with a dangerous fever. In 1762 ſhe had an eryſipelatous inflammation on her right thigh. In 1763 ſhe had ſeveral troubleſome ulcers on different parts of her body. In 1764 M. Morand preſented an account of her diſeaſe, together with her portrait, to the Academy of Sciences. In 1765 ſhe took ſome doſes of manna, which brought on a diſpoſition to vomit that laſted nine months, and determined her not to take any more medicines. In 1768 ſhe had an eryſipelas on her right arm. In 1772 her menſes began to be irregular. In 1775 her appetite, which till then had been good, began to fail, and in the month of December of that year ſhe died. On diſſection her bones were found to be ſo thin and fragile, that on preſſing one of the knees with a finger, the head of the tibia was broken, and the finger paſſed into the cavity of the bone. The marrow was in a conſiderable quantity, but in a vitiated ſtate. The cartilaginous part of the bone was wholly deſtroyed, the earthy part remaining almoſt alone, ſo that this diſeaſe ſeemed to be totally different from that of Supiot's wife. The muſcular fleſh was almoſt entirely deſtroyed, excepting that of the glutæus maximus, the abdominal muſcles, and the deltoid, which were the only ones ſhe uſed. In the lower extremities the tendons only remained, and theſe were very ſtrong, ſtiff and ſtretched. In lieu of muſcles there was only cellular membrane, in which the traces of muſcles and blood-veſſels were with difficulty diſtinguiſhed.

The heart, like the fleſh of all the other muſcles, was without conſiſtence, and eaſily torn. The lungs were flaccid, and the liver, which was enlarged, and of a ſoft, pulpy conſiſtence, ſeemed to be kept together only by its membranes. A ſchirrous concretion was obſerved in one of the ovaries, and the doublings of the peritonæum were filled with ſchirrous tumours.

M. Saillant ſuppoſes this curious diſeaſe to have been a *plica polonica*. The ſkeleton of the woman is in the poſſeſſion of the Faculty of Phyſicians at Paris.

*Medical Chemiſtry.*—1. *New Proceſſes for preparing Martial Æthiops. By* Meſſieurs Maret, Darcet, Croharé, and Joſſe.——2. *Accounts of the Medical Properties of fixed Air in a Cancerous Caſe, by* Dr. Targioni of Florence, *and in Caſes of Putrid Fever and Phthiſis, by* M. Maret. None of theſe accounts are ſufficiently ſatisfactory.——3. *A Method of preparing*

*Emetic*

*Emetic Tartar.* *By* M. Durande of Dijon.——4. *A Method of preparing Calomel.* *By* M. Bailleau, Apothecary at Paris. This procefs confifts in mixing the corrofive fublimate and water into a pafte, and triturating it with crude mercury till the latter is extinguifhed, which is generally effected in half an hour. The mixture being then digefted in a fand-bath with a gentle heat, it becomes white, and forms a very mild mercury, which requires only a fingle fublimation to be perfectly pure.——5. *A new Method of preparing an Extract of Opium.* *By* M. Joffe. This extract is faid to contain only the fedative virtue of the opium. It is prepared by ftirring it in a veffel with warm water, and repeatedly pouring on it frefh water till the latter ceafes to be coloured. The coloured water is then to be evaporated to the confiftence of an extract, which is to be given in dofes of two grains. What remains undiffolved is faid to be the glutinous part of the opium.——6. *A Manner of preparing the mealy part of Potatoes.* *By* M. Gallot.——7. *Remarks on the Spirituous Fermentation of Milk*, *by* Dr. Spielmann.——8. *Experiments on Urine*, *by* M. Bucquet. Thefe experiments were made on the urine voided by Mrs. Souchot, after fhe had undergone the fection of the Symphyfis Pubis, and which appeared to be very different in its smell from that which has remained any time in the bladder.——9. *A Chemical Analysis of Cantharides and some other insects, by* M. Thouvenel.——10. *Analyses,* by different writers, of the Mineral Waters of *Chateldon* in Bourbonnois (chalybeate); *Saulchoir* near Tournay (chalybeate); *Digne* in Provence (hot); *Sainte Reine* in Burgundy (impregnated with fixed and volatile alkali, ol. petrolei and earth); *Manjolet* in Roussillon (hot, sulphureous and chalybeate); *Bouillants* near Avranches (acidulous chalybeate); *Roye* (chalybeate); *La Craute* in Burgundy (sulphureous); *Saint Santin* near Aigle, *Orlienas* near Lyons, and *Rainsy.* The three last are chalybeates.

*Botany, and the Natural History of Drugs.*——1. *Of the Use of the* Faba Sancti Ignatii *in Intermittents. By* M. Four de Bourieu. The author recommends this remedy in cases that resist the bark. To adults he gives twelve, to children only six grains for a dose.——2. *The Root of* Timac *recommended in Dropsies. By* M. Gerard, physician at St. Domingo. This root is given in decoction. The plant it belongs to is not described.——3. *A Case of Dropsy cured by a Decoction of* Galega floribus cæruleis, B. *By* M. Moulien,

Moulien, phyfician at Rennes.——4. M. Durande, of Dijon, recommends the leaves of the *Ilex Aquifolium* L. or *Holly-tree*, in intermittents, in preference to the Peruvian bark. They are directed to be given dried and powdered in a dofe of a drachm before the fit.——5. M. Aymel recommends a tincture of *Ifatis* or *Woad* as a remedy in Scurvy.——6. *Remarks on Smutty Rye and Wheat. By* Father Cotte and M. Parmentier.

*Medical Philofophy.*—1. *A Method of preferving Water frefh at Sea. By* M. la Peyre. This confifts in wafhing the cafks with lime water, and impregnating the water with lime and acid of vitriol.——2. *An Account of the Effects of Lightning. By* M. Brillonet. This gentleman was ftruck with lightning, but without being materially hurt by it. Six weeks afterwards he perceived that the fciffars and lancets he had in his pocket at the time of the accident had acquired a powerful magnetic quality.——3. *A Cafe of Afphyxy on opening a Well. By* M. Bonami, phyfician at Nantes.——4. *Remarks on the Adulteration of Cyder, and the means of difcovering it. By* M. Bucquet.——5. *An Account of the Inflammable Air of a Well. By* Father Cotte.——6. *Experiments relative to*

*the*

*the Condensation of Mercury and Spirit of Wine. By* the same. These experiments prove, that mercury is more susceptible of condensation, but less of dilatation than spirit of wine.

Having given this summary view of the first or Historical part of the volume, we proceed now to the second part or Memoirs. The first article in this division is *an Account of the Diseases that prevailed at Paris in* 1775 *and* 1776. *By* M. Lorry. This paper contains a very accurate history of the epidemic catarrh which began to appear at Paris about a month after the autumnal equinox of 1775, and did not wholly disappear till the following spring. A similar disease, we are told, prevailed at the same time among domestic animals. The most general symptoms were an acute pain in the head and limbs, and a copious expectoration of a clear, limpid mucus. Several cases occurred in which, by neglect of regimen, exposure to cold, or some other irregularity when the disease was nearly over, the symptoms were renewed. Many gouty patients who had a regular fit, escaped the epidemic, and others who were subject to wandering gout and rheumatism, felt it only by an increase of pain. It was observed, that women subject to profuse fluor albus were less liable to it than others.

In fome inflammatory habits the difeafe was attended with eryfipelas. The meafles, and indeed all the difeafes which immediately fucceeded this epidemic, feemed to partake of it. In one cafe of meafles, in a female patient, our author faw the difeafe interrupted, as it were, by a flux of the menfes, and when that ceafed, refuming its former violence. In fome cafes he faw a painful hardnefs of the liver, brought on by the meafles, and carried off by a bilious diarrhœa. ——2. *An Account of an Epidemic that prevailed at Touloufe in the Autumn of* 1772. *By* M. Gardeil, Profeffor of Phyfic at Touloufe. This epidemic was a remittent fever, complicated in many patients with dyfentery. It began to appear in July, and continued till the end of December. During this period, 2782 perfons were admitted at St. James's, which is the principal hofpital in Touloufe. Of this number 306, or 1 in 3, died. The average number admitted in the fame fpace of time in ten preceding years, was only 1034, and the average number of deaths 196, or 1 in 6. ——3. *Defcription of a malignant Epidemic Fever, which prevailed at Coutances and its environs in* 1772 *and* 1773. *By* M. Bonté, phyfician at Coutances.——4. *A Memoir relative to Sologne. By* Abbé Teffier. This effay contains many interefting tereſting obſervations on the natural hiſtory, health of the inhabitants, &c. of this province. ——5. *A Memoir relative to Lorraine. By* M. Jadelot.——7. *An Account of the bad effects produced by the Miaſmata of putrid Animals, and of a new Method of Treatment ſucceſsfully employed in thoſe Caſes. By* M. de Laſſone. In 1749 a diſorder prevailed among the horned cattle, which carried off a great number of cows in the environs of Paris. Many of theſe were buried in the neighbourhood of the convent of *l'Enfant Jeſus*, to which our author was at that time phyſician, and the air ſoon became infected. At the beginning of winter thirty ladies of the convent, and ſeveral of the ſervants, were attacked either with dyſentery or putrid fever, or both. The progreſs of the infection was ſtopped by the Police, who gave orders to bury the carcaſes of the cows deep, and to cover them with lime. The new mode of treatment purſued by our author in theſe caſes, conſiſted in a liberal uſe of bark clyſters.——8. *An Eſſay on the Hydrophobia. By* M. Andry. In this paper, which has been publiſhed ſeparately, the author has collected almoſt every thing that has been ſaid on the ſubject by different writers. It is divided into three parts. In the firſt he treats of the ſponta-

ſpontaneous hydrophobia, of which he quotes numerous inſtances from authors, but none from his own obſervation; in the ſecond he deſcribes the contagious hydrophobia; and the third and laſt part relates to the method of treating it. The various remedies that have been at different times propoſed for the cure of this diſeaſe, are fully deſcribed.——9. *Obſervations on the Leproſy of Martigues. By* M. Vidal. This place formerly was famous for the number of patients in this way (ſee *Med. Obſ. and Inq.* Vol. I.) but at preſent there are but few. Our author's obſervations prove that this diſeaſe is hereditary, but not contagious. The common people, we are told, live in the ſame manner here now that they did a century ago, their food conſiſting chiefly of fiſh; but they are more cleanly in their houſes and perſons, and are better cloathed. The population too, from 15 or 18000, is reduced to 7 or 8000.——10. *An Account of a Diſeaſe called* Crinons, *which attacks new born Infants at Seyne in Provence. By* M. Baſſignet. This diſeaſe, which is ſaid to be peculiar to the town of Seyne and its neighbourhood, attacks almoſt all the new-born children. Authors call it *Crinons* or *Comedons*. In the place itſelf it is called *Cées*, a corruption of *Ceddés*, a provencal word that ſig-

 nifies nifies *a briſtle.* It appears in many caſes within twelve hours, in others not till a month after birth, and ſometimes, tho' rarely, at a more advanced age, of even twelve or more years. The ſymptoms are deſcribed to be a violent itching, which is increaſed by the heat of the bed, and prevents ſleep; a continual agitation; impoſſibility of ſucking, the child's tongue not being able to accommodate itſelf to the nipple; and laſtly a hoarſeneſs, and gradual extinction of the voice. Of all theſe ſymptoms the laſt is conſidered as the moſt certain, ſo that by the weakneſs of the child's cries, and the alteration in its voice, they judge of the degree of the diſorder. As ſoon as it is obſerved they proceed to the cure, which conſiſts in frictions by women of the country, who are ſo accuſtomed to this diſeaſe that they ſeldom call in either a phyſician or ſurgeon. Theſe frictions are made on different parts of the body, according to the three ſtates of the diſeaſe, which are ſometimes diſtinct, at others complicated. In the firſt, to a diminution of voice is joined an inability to ſuck. This, we are told, requires frictions at the upper part of the ſternum, neck, cheeks, and about the jaws and temples. If the child's tongue is at liberty, and yet he is ſtill unable to ſeize the nipple, his arms or fingers at the ſame time feeling tenſe, this is the ſecond ſtate of the diſeaſe, and requires frictions on the fore arm. The third is known only by the change in the voice, and is cured by rubbing the arms, ſhoulders, back, and calves of the legs. The mode of friction is as follows: the woman wets her hand with ſaliva, and rubs the ſkin of one of the child's arms, for inſtance, along the extenſor muſcles, till ſhe feels a conſiderable roughneſs. She then quits this arm and begins with the other, rubbing always in ſmall circles, and conſtantly in the ſame direction. Nothing particular is obſerved in the ſkin previous to theſe frictions. Some of the moſt experienced women, however, ſpeak of a ſort of tenſion which gives way to rubbing. In many caſes where this practice has been neglected, the child, we are told, has been carried off by convulſions or diarrhœa. In ſome ſubjects, a ſpecies of dark, rough hairs, not longer than the tenth of an inch, and in others, little ſubſtances reſembling very fine red hair, not quite ſo rough as the former, and furniſhed with a minute bulb at their extremity, appear on the ſkin, and terminate the diſeaſe. This circumſtance it is that gives name to the diſeaſe. Our author ſpeaks of a girl ten years old, who, after having been for ſome time ill, and taking different medicines, at length tried frictions in the manner juſt now deſcribed, and theſe brought out a prodigious quantity of dark coloured, rough hairs, after which the patient got well.—This extraordinary diſeaſe is treated of at ſome length by the learned M. Lorry, in his *tractatus de Morbis Cutaneis*, lately publiſhed: it is likewiſe mentioned by Ambroſe Paré, whoſe account of it, as it is very conciſe, we ſhall here tranſcribe from Johnſon's tranſlation of his works (*page* 215.) "The mention of the *Dracunculi* calls to my "memory another kind of abſceſs, altogether "as rare. This our Frenchmen name *Cridones*, "I think *a Crinibus*, *i. e.* from hairs. It chiefly "troubles children, and pricks their back like "thorns. They toſs up and down, being not "able to take any reſt. This diſeaſe ariſeth "from ſmall hairs, which are ſcarce of a pin's "length, but thoſe thick and ſtrong. It is "cured with a fomentation of water more than "warm, after which you muſt preſently apply "an ointment made of honey and wheaten flour; "for ſo theſe hairs lying under the ſkin are "allured and drawn forth; and being thus "drawn, they muſt be plucked out with ſmall "mullets. I imagine this kind of diſeaſe was "not known to the ancient phyſicians."——

11. *Expe-*

11. *Experiments on the Manner in which Animals are affected by different aeriform mephitic Fluids, and on the means of remedying thoſe effects. By* M. Bucquet. About two hundred animals (quadrupeds, birds, or frogs) were ſuffocated for the purpoſes of this paper, in fixed air, fumes of charcoal, or inflammable air.——12. *An Eſſay on the Miliary Fever, containing a Deſcription of the Symptoms, Varieties, and Complications of that Diſeaſe. By* M. Barailon. The miliary eruption is deſcribed in this paper as being always preceded by profuſe ſweating, and its diſappearance in certain caſes is attributed by our author to "the "taking the patient out of bed, or giving him "an immoderate quantity of acidulated drink, "by which means we check the fever, which is "oftentimes too inconſiderable to be able to put "the morbific matter in motion." This erroneous theory ſhews that he is a friend to a heating regimen.——13. *Remarks on Corrupted Water. By* M. Mauduyt. This eſſay contains ſalutary cautions againſt the uſe of ſtagnant and corrupted water.

[ *To be concluded in our next.* ]

VIII. *An*

